# When parents won’t vaccinate their children: a qualitative investigation of australian primary care providers’ experiences

**DOI:** 10.1186/s12887-017-0783-2

**Published:** 2017-01-17

**Authors:** Nina J. Berry, Alexandra Henry, Margie Danchin, Lyndal J. Trevena, Harold W. Willaby, Julie Leask

**Affiliations:** 1 Sydney School of Public Health, University of Sydney, Edward Ford Building A27, Sydney, NSW 2006 Australia; 2 Lismore Base Hospital, 60 Uralba St, Lismore, NSW 2480 Australia; 3 Murdoch Children’s Research Institutes, Royal Children’s Hospital, 50 Flemington Rd, Parkville, 3052 VIC Australia

**Keywords:** Vaccination, Iimmunisation, Communication, Vaccine-hesitance, Consultation, Primary healthcare provider

## Abstract

**Background:**

Increasingly, the experiences and perceptions of parents who decline vaccination are the subject of investigation. However, the experiences of clinicians who encounter these parents in the course of their work has received little academic attention to date. This study aimed to understand the challenges faced and strategies used when general practitioners and immunising nurses encounter parents who choose not to vaccinate their children.

**Methods:**

Primary care providers were recruited from regions identified through the Australian Childhood Immunisation Register (ACIR) as having higher than national average rates of registered objection to childhood vaccination. Interviews began with an exploration of provider experiences with parents who accept, are hesitant towards, and who decline vaccination. Participants were asked specifically about how they addressed any difficulties they encountered in their interactions. Thematic analysis focused on encounters with parents – challenges and strategies.

**Results:**

Twenty-six general practitioners (GPs), community and practice nurses (PNs) were interviewed across two regions in NSW, Australia. Providers’ sense of professional identity as health advocates and experts became conflicted in their encounters with vaccine objecting parents. Providers were dissatisfied when such consultations resulted in a ‘therapeutic roadblock’ whereby provider-parent communication came to a standstill. There were mixed views about being asked to sign forms exempting parents from vaccinating their children. These ranged from a belief that completing the forms rewarded parents for non-conformity to seeing it as a positive opportunity for engagement. Three common strategies were employed by providers to navigate through these challenges; 1) to explore and inform, 2) to mobilise clinical rapport and 3) to adopt a general principle to first do no harm to the therapeutic relationship.

**Conclusions:**

Many healthcare providers find consultations with vaccine objecting parents challenging and some, particularly more experienced providers, employ successful strategies to address this. Primary care providers, especially those more junior, could benefit from additional communication guidance to better the outcome and increase the efficiency of their interactions with such parents.

## Background

Primary care professionals encounter a variety of challenges in their provision of vaccines to children. These providers must keep up-to-date with increasingly complex vaccination schedules; manage busy clinic schedules; assuage distress about injection pain; and address parental ambivalence about, or even opposition to, vaccination.

While 92% of children living in Australia are fully vaccinated there is a persistent group, estimated at 3.3%, who decline completely, select out or delay vaccinations (vaccine objecting parents). However, these national figures mask regional variations in vaccination objection with some regions recording vaccine objection for up to 14% of resident children [3, 15].

Since 1998, parents or caregivers have been required to demonstrate that their children aged five years or under are fully vaccinated for age, in order to access federal government family assistance payments, and in some jurisdictions, entry to pre-school or kindergarten. Exemptions to this requirement have been available on the grounds of medical contraindication or personal belief. These exemptions were granted if parents provided a form signed by themselves and a vaccination provider (usually a General Practitioner or Registered Nurse). Exemptions on the grounds of personal belief (including religious beliefs), called “Conscientious Objection”, required a provider to declare that they had “*explained the benefits and risks associated with vaccination to the parent or guardian…and informed him/her of the dangers of not vaccinating”*. This category of exemption was revoked on 31 December 2015.

This qualitative study aimed to explore vaccination providers’ own accounts of consultations with parents who decline some or all vaccinations for their children; the challenges that arise and the strategies providers’ employed to address those challenges. The magnitude of the challenge presented to vaccination providers by parents who decline vaccination for their children, is evidenced by providers’ own observations (admissions) that these consultations frequently degenerate into adversarial encounters, even though they are aware that engaging with parents in this way is almost invariably fruitless [20].

Nevertheless, primary care providers are known to influence parents’ decisions about their children’s health generally [9], and exert significant influence on parents’ vaccination decisions, both positively and negatively [4–6, 19]. Importantly, parents who have reversed a decision to decline vaccination for their children identified ‘information or reassurances’ from a health care professional as the most important driver of this reversal ([14, 23].

The results reported here are a subset of a larger investigation that also sought providers’ feedback on the application of the Vaccination Communication Framework [21]. This study forms part of a multi-study investigation of the perceived vaccination information and communication support needs reported by parents and primary health care providers. The results of this investigation will inform development of a package of communication and information resources and strategies designed to assist health care providers to proactively and positively address vaccine rejection and hesitancy in primary care.

## Methods

### Study design/ approach

Qualitative in-depth interviews were used to explore the experiences and practices of childhood immunisation providers (General Practitioners and Registered Nurses giving immunisations) working in two regions of NSW where Conscientious Objection (CO) exemptions to childhood vaccination are higher than the national average and full vaccine coverage by one year of age is lower. An additional two providers, based outside these areas, and who expressed interest in participating in the research via social media were also interviewed.

Interviews were structured with reference to parental positions described by Leask and colleagues [21] Although providers’ experiences with parents who accept vaccination were explored, this paper focuses on their experiences with the smallest but most challenging group of parents; those who present as unwilling or not yet ready to vaccinate their children (Selective/Delaying or Declining parents). A brief, thematic interview guide was developed to ensure consistency between interviewers (Appendix). Ethical approval for the study was granted by the Human Research Ethics Committee of the University of Sydney.

### Recruitment and sampling

Primary health care providers were recruited to participate in the research using a mixed recruitment strategy, which combined purposive sampling (identifying a population of health care providers likely to encounter parents who decline vaccination) and intensity sampling (including respondents with a particular interest in vaccination).

Using the latest available data recorded for the Australian Childhood Immunisation Register (ACIR), we identified two local health districts (LHDs) in which very low vaccine coverage was observed amongst a single quarterly birth cohort, and in which registered CO exemptions were higher than the national average of 1.77% [1]. One of the LHDs was situated in a rural area and the other in an urban area. Around 83% of children born between the 1st January and the 31st March 2002 living in these areas were fully immunised at age two, compared with 92% nationally. In the rural LHD, CO exemptions had been recorded for 6.5% of the cohort. However, geographically defined sub-groups within this LHD were observed to have recorded exemptions more than four times this rate. CO exemptions in the urban LHD were lower (0.9% overall), ranging between 0.6% and 4.4%.

Practices located within these LHDs were identified and initial contact made by mail addressed to the Practice Manager (in rural area) or email (in the metropolitan area) via the list belonging to the primary care region’s organisational unit, then named a “Medicare Local”.

General Practitioners (GPs), Practice Nurses (PNs) and Community Nurses (CNs) were invited to contact the research team if they were interested in participating in the study. In NSW, 85% immunisations are given by a nurse or doctor in general practice with 12% by public community health clinics [2]. In the rural region, a researcher followed written invitations with face-to-face contact (visits) to offer further information about the study. Providers who expressed interest in participating were then contacted by telephone or email to schedule interview appointments.

Intensity sampling is known to increase access to interesting and divergent responses, broadening the scope of the enquiry, providing richer insights than would otherwise be possible [22]. These cases can also be a source of thematic triangulation. Mixed recruitment strategies, combining purposive intensive sampling, have been used elsewhere [20, 22].

All respondents were interviewed in the initial tranche of interviews. Initial analysis informed a second, purposive round of recruitment, to ensure the range of immunisation provider types were represented. Recruitment ceased when the research team judged thematic saturation had been achieved.

### Data collection

Interviews were conducted in two local government areas between September 2013 and December 2014 in meeting or consultation rooms at various locations convenient to participants. Interviews were conducted by four trained researchers, all of whom had prior experience conducting research interviews and none of whom had prior relationships with the respondents. Digital audio recordings were made and brief supplementary field notes were taken. To preserve fidelity, the senior author trained all interviewers, listened to initial interviews and offered feedback or further instruction as required. Interviewers moderated their data collection strategies by listening to a sample of one another’s audio files, identifying variances, discussing and making minor modifications to their strategies as required.

Interviewers introduced themselves by describing their roles at the university and their interest in vaccination communication research, at recruitment and prior to commencing the interviews. Each participant was interviewed only once and interviews lasted approximately one hour. Participants were offered the opportunity to check their transcripts but none accepted the invitation. Digital audio files were uploaded to a secure server, whereon they were transcribed by a commercial transcription service and deleted from remote devices.

### Analysis

Analysis followed a grounded theory approach [12, 13]. After the first tranche of interviews, transcripts were read and re-read with early codes noted (open coding). Two other researchers independently read a sub-sample of transcripts selected to capture the variation within the sample. A coding workshop was convened to discuss emergent themes and agree on a coding framework. These early themes informed recruitment of respondents for the second tranche of interviews. The second tranche of transcripts was read and the coding scheme was modified slightly to account for further commonalities and variations in participant responses. The data were coded by hand.

In addition, responses given by the two providers who practice outside the target LHDs, were interrogated. This strategy aimed generate insight into the question of whether the accounts given by providers working in low-coverage communities were typical or reflected phenomena shaped by the peculiarities of particular, geographically defined communities.

## Results

### Respondents

A total of 26 interviews were completed and analysed. Seventeen participants were GPs and nine were practice or community nurses. There were equal numbers of respondents (*n* = 13) from the urban and rural LHDs. Seniority and experience varied widely amongst respondents. Six respondents had less than three years’ experience. The remainder had been in their roles for at least three years; some for more than twenty. The majority of providers (24/26) worked in general practices and two worked for community health organizations offering childhood vaccination clinics. All providers who approached the research group in response to recruitment activities were interviewed. The responses given by the two self-recruited providers accorded closely with those offered by providers working in highly vaccine resistant communities.

Our primary finding was that a clinical encounter with a non-vaccinating parent posed significant *challenges* to healthcare providers and to the provider-parent relationship, which they reported using a variety of *strategies* to address. In this paper, we explore how declining vaccination was often viewed as conflicting with providers’ *professional identities*, sometimes seen to generate a *communication roadblock*, which hampered effective communication with parents and placed the desired clinical outcome in jeopardy. Thereupon we describe a variety of communication strategies providers reported employing to *explore and inform* parents who decline vaccination for their children, and note their commitment to *mobilising clinical rapport* as a powerful tool to promote vaccination. Finally, we illustrate providers’ application of the principle, *primum non nocere* - *first, do no harm* – to prioritising and protecting therapeutic relationships in the service of child health.

### Experiences

#### Professional identity

Non-vaccinating parents were often perceived as challenging respondents’ legitimacy as health advocates and medical experts. Respondents described experiencing internal conflict, driven by a sense that they were bound by a set of moral and professional obligations to the health and wellbeing of the child before them and to their communities through upholding vaccination coverage; and that these obligations contained apparent contradictions that were difficult to resolve. Parents who decline vaccination for their children were seen to frustrate practitioners’ capacity to provide appropriate medical care to that family, while simultaneously presenting for that medical care. Many respondents reported feeling very frustrated, even angry, during these encounters.

*I felt the pressure of the responsibility to the child (GP12)*

*It just really angers me that there are whole populations of kids here whose kids aren’t protected, because other parents have been irresponsible (GP4)*

Some respondents described experiencing parents’ resistance to vaccinating their children as a direct challenge to their identities as trusted authority figures. Some perceived parents’ questioning as casting doubt on their professional judgment and their integrity. Some described decling parents as expressing a fundamental mistrust of established scientific, medical or pharmaceutical evidence, or saw it as unwillingness on the part of parents to trust the medical establishment. A few respondents described declining parents expressing preferential trust in alternative epistemologies or practitioners, including traditional, complementary, alternative or ‘natural’ medicine and peer narratives.

*I think there is quite a strong pushback against medical establishment. So the doctor used to be an authority figure and the doctor used to be someone who could not be questioned (GP14)*

*They’ll go to the naturopath before they come to the doctor, or they’ll go to the chiropractor before they come to the doctor. Those people will often tell them ‘don’t get vaccinated,’ and so they’ve heard those things from people they trust more than the doctor (GP7)*

#### Signing the vaccination objection form

We observed a spectrum of attitudes amongst the respondents towards signing parents’ exemption forms. Although none reported refusing to sign the form, some found doing so presented uncomfortable internal conflicts between professional duties (to provide appropriate medical care to the child and family; to protect the community), their contractual obligations (to the Australian Government as authorised providers of Medicare services or to state governments as employees), and their concern to maintain functional therapeutic relationships with these parents. These providers felt they had been co-opted to perform a function that was outside of, or actually conflicted with, their usual scope of practice. They felt they had been conscripted to facilitate access to financial incentives through government legislation, effectively rewarding parents for failing to comply with an effective and important public health initiative. These respondents expressed discomfort with appearing to condone a parent’s decision to decline vaccination for a child and therefore place them and others in the community at risk of infectious diseases.

*The issues are in the sense it’s about payment, so that’s the reason why they’ve come. Why should we be drafted in to sign a form that’s actually about payment? Once you’ve got a monetary issue, it’s very hard to have a conversation about (vaccination). So why is it fair for doctors to be asked to do that? It is an ethical dilemma that the government has created (GP6).*

Junior practitioners were particularly conflicted when trying to balance their obligations to the individual interest of the family with the risk to the broader community that non-vaccination posed and the exemption form represented. Occasionally, a provider felt s/he was being asked to participate in a policy that offered parents (financial and social) incentives to decline vaccination – or at least neutralised disincentives. A few expressed the view that parents who actively declined vaccination ought to be denied access to childcare and tax benefits, articulating a moral connection between childhood vaccination and entitlement to publicly funded health and welfare.

*If they don’t believe in the system, why should they benefit from the system? (GP14)*

Other respondents saw the form-signing process as an opportunity to engage with parents’ decision-making process and address their concerns. In particular, more experienced practitioners tended to be more comfortable with process. A few practitioners saw it as formalising a legal obligation for them to present the risks of non-vaccination. Others described using it as a tool to facilitate engagement with a highly hesitant parent. Agreeing to sign the form was seen as an expression of respect for the parents’ decision, which then opened the way for continuing discussion about vaccination. Several GPs reported using the form as a vehicle for advocacy for their patients, expressing a belief that non-vaccinated children should be entitled to the same access to childcare and tax benefits and as vaccinated children.

*I’m happy to sign it, because I think first of all it’s good to engage with (vaccine refusers) and secondly I don’t think they should be discriminated against” (GP1).*

#### Communication roadblock

Most respondents reported feeling frustration during their encounters with non-vaccinating parents who were unwilling to engage in discussion. They described these parents as intractable and felt their efforts to persuade them to consider vaccinating their children were largely fruitless. They used emotive language to describe the parents who object to vaccination (‘difficult’, ‘challenging’, ‘ignorant’, ‘closed’, ‘no common sense’, ‘shut down’, ‘impossible’, ‘resistant’) and their own experiences of consultations with them (‘hurt’, ‘stressed’, ‘I give up’, ‘waste of time’, ‘pointless exercise’).

*They say ‘I know what you’re going to say, I know about the science behind it, I’ve read everything, you’re not going to change my mind, I’m not vaccinating my child’ (GP9)*

*Every time you come to a brick wall, it gets harder and harder (RN1)*

Some described finding themselves in a kind of clinical impasse during these consultations and feeling powerless to resolve the situation.

*Their answer is ‘no not doing that, see you later’ that’s it. That’s the most difficult, I mean what do you do with that?’ (GP5)*

Some believed that some consultations were so emotionally charged or laden with potential for conflict that they risked damaging the therapeutic relationship. One respondent expressed a desire to avoid the impasse thus,

*‘If they’re immunised, I breathe this huge sigh of relief, because I just don't have to go there’ (GP6)*

Most providers felt it was not possible to have an effective discussion with parents who object to vaccination within a standard 15-min consultation. Many reported that their communication skills were compromised on becoming flustered because they were trying to rush through the parent’s questions and were conscious of their obligation to avoid delaying subsequent patients.

### Strategies

To manage these very challenging consultations with vaccine objecting parents, providers described employing three common strategies: exploring and informing, mobilising clinical rapport and protecting the therapeutic relationship, which was understood to constitute an application of the long-cherished ethical mandate, *above all do no harm.*

#### Explore and inform

Respondents described attempting to explore parents’ reasons for vaccine objection and then offering credible information in response. Discussions could be broadly categorized into; 1) concern-based (i.e. countering a specific worry, such as whether vaccines are implicated in the development of autism) 2) risk-based (i.e. whether risk of contracting a VPD or complication is important, risk of travelling with a non-vaccinated child) or 3) knowledge-based (i.e. how the immune system works, benefits of herd-immunity, the practicalities of non-vaccination with regards to childcare/school entry and payments).

Many respondents found it helpful to first engage in enquiry through asking permission to enter the conversation, open-ended questioning or respectfully challenging a belief (Table 1). Those who reported successful interactions with parents described using supportive language such as ‘dispelling fears’ ‘reassurance’ and ‘alleviating worry’ to describe their approach. These clinicians reported being vigilant for cues about their patients’ health literacy or receptivity to information, and they were cautious to avoid overwhelming parents with facts or scientific research.

**Table 1:** Examples of enquiry described by providers for exploring parental vaccine-objection

Asking permission	“Would you mind telling me what in particular you’re worried about?”
	“Do you mind if I bring up vaccination at your next visit?”
Open-ended questioning	“How are you feeling …?”
	“What would you like to do?”
Respectfully challenging a belief	“That’s interesting, what do you mean by that? ”

*You don’t want to cloud the issue. You work out what your major message is and you make sure that's the last thing you say. Because if you give them too much information, the whole brain shuts down (GP16)*

However, a few providers described more confrontational strategies. Some reported dismissing parents’ concerns entirely.

*Well that's rubbish, you do know that?*

Others reported attempting to motivate parents into accepting vaccination with appeals to fear.

… *those parents who I cannot persuade with reason, I used to give them a video of children choking and dying of whooping cough, dying of measles, meningitis, dying of diphtheria. Cemeteries full of kids dying of tetanus*.

And some described reframing parents’ concern for their children’s wellbeing to incite guilt.

*Your little baby who could potentially get a fatal disease is not protected. How would that make you feel?*

Providers commonly referred to government-issued resources or their local public health units for information with which to address parents’ concerns. Some described referring to resources with the parent during consultation time or send them home with written information. Less commonly described approaches included examining each specific vaccine ingredient on the vaccine information leaflet with the patient. One spoke of inviting a naturopath into the consultation room to facilitate a balanced discussion.

#### Mobilising clinical rapport

Gaining a parent’s trust was viewed as a crucial tool in navigating around the communication roadblock. Providers were keenly aware that building a therapeutic relationship was the first step in moving a parent towards immunisation and they employed several strategies towards this goal.

*Rapport is extremely important and I know that when I get someone who’s got some concerns about vaccination, if I can’t establish that therapeutic rapport with them, then it’s going to be difficult (GP5)*

Listening respectfully, avoiding judgemental language, offering more time and being mindful to avoid criticising or pressuring the parent were commonly referenced methods to *express empathy*. Experienced providers reporting positioning themselves as parents’ allies, primarily by agreeing to sign the form (meeting the parent’s perceived need) before attempting to address their concerns.

*I’ll say to them ‘You’re obviously a really great Mum, you really care about your kids’ --- I’ll say something affirming, something positive to the Mum so you let them know that you’re on their side – even though I’m 100% ideologically disagreeing with the decisions they’re making (GP9)*

The notion of *reciprocity* was also important. One GP, who reported establishing a strong relationship with the family by supporting the unimmunised child through an illness, described finding it easier to broach the issue of vaccination and then to persuade the mother to consider vaccination:

*I think the mother honestly just appreciated that, and at some [point] we brought up immunisation and she said ‘well what did you think?’ and I said ‘well I do think it’s really important’. And it seemed as if from that, she said ‘right, okay’.(GP6)*

Several clinicians reported *re-framing* vaccination as being an action consistent with the parent’s strongly held views. For a parent concerned about vaccination as ‘unnatural’, some reported presenting it as a natural stimulator of the immune system. Similarly, some respondents suggested vaccination could be promoted as a socially conscious act and a unique responsibility of a ‘good parent’.

When it was clear parents would not vaccinate, some providers reported building rapport by *offering selective or delayed vaccination* as an alternative to the full schedule. This was seen as an opportunity to validate the parent’s concern while minimising harm. In some cases, the offer of selective or delayed vaccination was seen to serve as a ‘foot in the door’ – an initial transfer of decision-making power that opened the way for acceptance of other vaccinations, perhaps through demystifying the experience of vaccination.

*I start off and say ‘look … you don’t have to vaccinate for like that specific vaccination, you can vaccinate around it’. It’s classic - they start vaccinating with one, and then they end up saying ‘look just do it all’. And you end up doing everything (GP2)*

However, a few were very protective of the schedule, unwilling to deviate from it, either because it was not possible (many of the vaccines on the NIP are only available as multivalent vaccines), logistically difficult (requiring many more presentations to a clinic or surgery), or because it presented an unacceptable compromise.

*They just wanted to pick and mix and you know, we're just going, "Uh really? It's just what you're asking for is too difficult, it's too complicated, it doesn't exist, it's not possible (RN2)*

Several providers advocated offering an amended schedule, but admitted experiencing difficulty with this. They felt that they needed more information or that the process increased their workloads.

*I’d like a bit more information about maybe the middle road, because basically it’s schedule or nothing (GP1)*

#### First, do no harm

Ultimately, most respondents expressed the view that maintaining a positive provider-parent relationship is more important to the health of their patients than achieving vaccination. Providers generally prioritised keeping the parent engaged with healthcare, and in some cases this concern drove them to sign Vaccine Objector Forms. Some noted that maintaining a positive relationship in the short-term was essential in re-introducing the vaccination at a later date.

*I’m reading a lot of the time ‘will you come back and see me or do you just feel bashed? – because I’ve got to create a relationship of some sort so that they’ll still keep coming back. And not just about the immunisation conversation, but about anything (GP6)*

Respondents even described *standing down* from their pursuit of vaccination if they felt the therapeutic relationship was at risk. This was seen most frequently amongst more senior providers who appeared more comfortable with vaccine objector parents and less inclined to conflate persisting vaccine objection with professional failure. Some providers spoke of changing their goals for a consultation once they had accepted vaccine objection.

*So then- then you move into the harm minimisation strategy. So firstly, don’t lose the patient because I think if you become this obnoxious person, "well I'm not going to look after your* child *if you don’t immunise them", or, "you're a bloody idiot, you know, go and get a life", all you do is you lose the patient and you lose the opportunity to be of some use and some value (GP14)*

We found that nurses reported less involvement with vaccine objecting parents, either because consultations with these parents did not reach the vaccinating nurse, or because objecting parents rarely present to nurse-led vaccination clinics.

## Discussion

This study found that most GPs and immunising nurses found discussions with parents who decline to vaccinate their children to be challenging. They felt that these parents cast doubt on their personal and professional integrity, their authority as medical experts and their competence as communicators. These encounters also stimulated strong internal dissonance; many providers felt their professional obligations (to the child and the community) conflicted with the primarily bureaucratic function of exempting parents from complying with the recommended immunisation schedule. Some seemed to resent being called upon to sign the exemption form at all. Some providers managed this dissonance with begrudging acquiescence.

These results are consistent with previous research in Australia and in other countries [17, 20]. One third of American physicians surveyed in 2009 viewed parents’ disagreement with their recommendations as a lack of respect for their medical judgement and experience. Even so, fewer than half of them (15% of respondents) refused to treat children whose parents chose not to vaccinate them [16]. A later survey found 40% of physicians agreed that requests from parents to ‘space out’ children’s vaccinations reduced their job satisfaction [17].

In adapting to the challenges presented by parents who decline vaccines, some providers reported developing a variety of response patterns. In our study most reported prioritising the therapeutic relationship even in the face of their misgivings about engaging in the exemption process. They believed that doing so would secure future opportunities for engagement and minimise the potential for harm to the child. Sometimes this involved offering an alternative vaccination schedule. Although some respondents expressed an unwillingness to consider diverging from the immunisation schedule.

The practice of prioritising the therapeutic relationship and maintaining parents’ trust is consistent with advice from professional bodies including the Royal Australasian College of Physicians and the American Academy of Pediatrics, which recommends against refusing to treat the children of parents who will not vaccinate their children [8, 18].

The use of engaged exploration to identify parents’ concerns and address them is also consistent with current evidence indicating that parents who are highly hesitant about vaccinating are seeking more balanced information (rather than promotional material) about vaccination that address their specific concerns [11]. While more senior clinicians were more at ease in their encounters with declining parents and more confident in their responses, it is likely they would benefit from opportunities to periodically review and reflect on the effectiveness of their interactions with this group of parents in order to expand their suite of communication tools.

The policy and historical context to this study is relevant. The study occurred during a time of intense national debate about immunisation policy and vaccine objector provisions in Australia at a time when a major newspaper was actively campaigning to remove exemption from childhood vaccination on the grounds of conscientious objection in federal and state legislation [10]. Some opinion leaders in the medical media vocally objected to the form signing process and this joined a generalised chorus of public outrage about parents who choose not to vaccinate their children leading to bipartisan support for an amendment to the incentives legislation.

Some of our respondents appeared to resent being asked to sign parents’ CO forms, viewing this as a civic or bureaucratic function that lay outside their roles as medical professionals; a function that threatened their professional independence and conflicted with their professional obligations to the child and the community. Historically, the medical professions in Australia have been uncomfortable with performing any task that could be or become civil conscription, which is held to be a direct threat to their professional autonomy, and therefore their capacity to act in the interests of their patients. ([7]: 20-23) This position mirrors the principle of the Separation of Powers, which protects the Judiciary (legal professionals) from government interference and has been enshrined in the Constitution of Australia since 1946, when a referendum passed an amendment prohibiting any civil conscription of doctors. Thus, the discomfort expressed by our respondents may reflect a concern that their professional autonomy is under threat from a government policy requiring them to perform an action that may be construed by parents as condoning their decision to decline vaccination for their children.

In November 2015, the Parliament of Australia eliminated the provision for exemption to the requirement that children be fully vaccinated in order for their parents to be eligible for certain financial benefits. While some state governments may continue to require parents who decline vaccination for their children to formally register their objection in order to access childcare, most providers will receive fewer requests from parents to sign their exemption forms. In its place will be new pressure on GPs to provide a medical exemption for previously vaccine objecting parents or to establish complex catch up arrangements in order to comply with federal and state requirements. Hence, the challenge of dealing with parents who choose not to vaccinate their children will remain and most likely intensify for Australian primary care providers. Additionally, our study may provide insights for jurisdictions that continue to require a clinician to sign a personal belief exemption as still occurs in some US states.

### Limitations

This study was limited to 26 healthcare providers, with a higher proportion of GPs compared to nurses participating (17 GPs vs 9 nurses). This may have under-represented the views of nurses. However, as we have suggested, GPs are more likely to have the primary encounter with vaccine-objecting parents. Whilst many providers expressed difficulty with the form signing process, this requirement will be minimised in future interactions as only medical exemption forms will be required to access financial benefits, as both philosophical and religious beliefs will no longer be valid. This study explores providers’ accounts of their behaviour during consultations with parents who decline vaccination for their children. It does not measure or report actual behaviour; neither is it concerned with doing so.

## Conclusion

We have identified several common challenges encountered by immunising GPs and nurses during consultations with parents who decline some or all vaccinations. The providers we interviewed were often conflicted about being required to sign Vaccine Objector Forms. Some perceived this task as threatening their efforts to maintain fidelity to their professional obligations to the child and to the community. Some perceived it as a threat to their fidelity to the health of their patient (the child) and the community and a few perceived it as an opportunity to engage parents in a conversation. In Australia and other countries, jurisdictions may involve vaccination providers in bureaucratic processes designed to inform parents or discourage parents from choosing not to vaccinate their children. Although it is often suggested that providers need access to educational materials that address common misunderstandings about the risks and benefits of childhood vaccination, this study suggests that providers also need communication strategies. Such strategies enable them to engender trust and protect therapeutic relationships with parents who decline vaccination. Many experienced providers reported developing these strategies in-situ. Others are likely to welcome further guidance, particularly more junior professionals with less experience in vaccination.

Any system that mandates the interaction of vaccine-declining parents with providers (such as for government payments or school entry) should provide support for those providers. This support may take the form of legal or professional advice; checklists or discussion guides; and/ or decision tools designed to support parents’ decision processes by helping them to consider the implications of their decision in a systematic way. Adequate training in conflict management and specialised communication techniques appropriate to situations of non-adherence should also be made available to vaccination providers.

Understanding providers’ accounts of their own responses to parents who decline vaccination for their children has provided important insights into their communication support needs. Further research will investigate parents’ information and communication needs. Together, these data will inform the development of a suite of communication tools that will be refined and tested.

## Appendix

### Interview discussion guide

#### Introduction

1. We would like to hear about the experiences and challenges you face when talking to parents about vaccination. We’re particularly interested in hearing about your experiences with parents who are hesitant/have concerns re; vaccination.
2. We’ve chosen your clinic because we’ve identified your postcode through ACIR as having higher than average levels of conscientious objection to vaccination.
3. We know that HCPs are one of the most influential factors affecting a parents decision to vaccinate or not, so we’d like to talk to GPs and PNs like you who regularly communicate with parents about vaccination.

### Perceptions and challenges

- I’d like to know about the parents who you encounter and their attitudes to vaccination. Could you describe a ‘typical parent’ who you might see, and their attitudes towards vaccination?
- Experiences with;
  - ○ Acceptors.... typical processes, resources, how do gain consent
  - ○ Hesitant….what do you say? Resources used?
  - ○ Refusers…how do they come to the conversation, CO form procedures, feelings raised.

### Resources

- What do you wish you had to make the conversations easier?

### The role of vaccination in the community and public health

- Perceptions of vaccination in region – rates are acceptable or problem
- Strategies to achieve consultation goals

### Communication challenges and perceived needs

- Challenges and perceived needs in meeting challenges
- Specific resources used when consulting with parents on vaccination decisions
- Could you describe the process (and any resources or checklists) you use to agree/obtain consent.

### Vaccine-communication framework

*“The National Centre for Immunisation Research and Surveillance has developed an evidence-based set of guidelines to assist HCPs to communicate effectively with parents about the benefits and risks of vaccination to aid in their decision making. As you’re probably aware, when it comes to immunisation not all parents fit the same mould, so these guidelines are tailored to the parent’s attitudes on immunisation to enable the most effective and efficient transfer of information. The aim of having this framework is to have an easy-to-understand tool that can be tailored to your patient and used easily within the standard consultation time. We hope you gain initial feedback on the prototype so we can modify it to make it as helpful as possible”*

### Reactions to Vaccination Communication Framework

#### (Show framework materials)

- Initial thoughts on framework
- Fit of framework with practice and community needs
- Realism, feasibility and potential implementation
- Training methods *(Show VCF Materials)*
- Description of training techniques that have been used in other studies to implement similar evidence-based guidelines. *(Explain each of the cards)*
- Feedback to improve the VCF, or other training methods that might work

## Abbreviations

ACIR: Australian childhood immunisation register
CO: Conscientious objector
GP: General practitioner
LHD: Local health district
PN: Practice nurse
RN: Registered nurse
